# Stress and turnover intention among healthcare workers in Saudi Arabia during the time of COVID-19: Can social support play a role?

**DOI:** 10.1371/journal.pone.0258101

**Published:** 2021-10-07

**Authors:** Khalid Al-Mansour

**Affiliations:** 1 Department of Social Studies, College of Arts, King Saud University, Riyadh, Saudi Arabia; 2 General Administration for Primary Health Centers, Ministry of Health, Riyadh, Saudi Arabia; St John’s University, UNITED KINGDOM

## Abstract

The turnover intention of healthcare workers is a threat to the competence of health services, especially during COVID-19 time. This study aimed to investigate the association between stress and turnover intention among healthcare workers in Saudi Arabia and whether social support could affect this association. In this cross-sectional study, healthcare workers in primary healthcare centers in Saudi Arabia responded to an online questionnaire assessing their sociodemographic and occupational history, stress levels using the Perceived Stress Scale-10 (PSS-10), social support using the Multidimensional Scale of Perceived Social Support (MSPSS), and turnover intention within the next few months. Path analysis was conducted to assess the mediating effect of social support on the association between stress and turnover intention. A total of 1101 healthcare workers (242 physicians, 340 nurses, 310 paramedics, and 209 administrative workers) participated in this study. The path between stress and support had a significant standardized regression weight (-.34, p < .05). The path between support and turnover had a significant standardized regression weight (.08, p < .05). The standardized total effect of stress on turnover without the impact of support was significant (-.39, p < .05). The direct effect of stress on turnover with the presence of support was significant (-.36, p < .05). The indirect effect of stress on turnover with the presence of support was significant (-.03, p < .05). Thus, there is evidence to show that support mediates the relationship between stress and support. Stress is associated with turnover intention among healthcare workers in Saudi Arabia. Social support had a mitigating effect on the relationship between stress and turnover intention.

## Introduction

The World Health Organization declared coronavirus disease-19 (COVID-19) a pandemic. The pandemic has affected more than 100 countries since it started [1]. Within about a year and half of this declaration, the COVID-19 pandemic has resulted in more than 4.3 million fatalities worldwide and a vast economic crisis with millions of people losing their jobs. Most countries responded to the spread of pandemics by imposing lockdowns, suspending schools, applying strict measures for social distancing, and preventing public gatherings. These decisions have been shown to create a global status of stress [2–6].

Studies have shown that healthcare workers managing COVID-19 have been experiencing worse psychological issues such as stress than the public because they are more likely to get infected and transmit the infection to their relatives and friends [7–9]. One of the major consequences of stress among healthcare workers is that it may result in increased turnover [10]. High turnover of healthcare workers might lead to disastrous consequences of the international efforts to contain the COVID-19 pandemic.

A few studies conducted during the COVID-19 pandemic have indicated high turnover intention among healthcare workers, yet none of them studied the possible role of stress in enhancing turnover intention. One study conducted on 261 nurses in the Philippines revealed that the fear of COVID-19 was associated with job dissatisfaction and increased turnover intention [11]. In Egypt, a study conducted on 210 frontline nurses working at a fever hospital during the COVID-19 pandemic showed that most nurses had turnover intention due to stress, stigma, and job dissatisfaction [12].

A similar study conducted on 117 nurses managing patients with COVID‐19 in Pakistan displayed that anxiety and the perceived threat of COVID-19 resulted in nurses’ turnover intention [13]. Another study conducted on Peruvian healthcare workers showed increased turnover intention during the COVID-19 pandemic [14]. All these studies were conducted in different countries and people with cultural backgrounds, which add value to this study to fill the knowledge gap of this issue in Saudi Arabia.

Recent studies have demonstrated several associations between the pandemic and stress among healthcare workers managing COVID-19. Social support was the major contributor to stress levels among healthcare workers in Egypt and Saudi Arabia during the COVID-19 [4]. Another study conducted on healthcare workers from China during the early months of the COVID-19 pandemic revealed that social support was strictly related to better mental health [15]. On the other hand, there is a negative association between social support and turnover intention [16].

Indeed, the pandemic was inversely associated with stress among healthcare workers [10, 15, 16]. It could be hypothesized that social support may minimize the impact of stress among healthcare workers on their turnover intentions during the COVID-19 pandemic. However, to date, the possible association between stress and turnover intention among healthcare workers in Saudi Arabia during the COVID-19 pandemic has not been studied. Especially, since the physical meeting with others was restricted as a result of COVID-19 pandemic. Also, whether social support could attenuate this association has not been investigated. Herein, this study investigated the effect of stress on turnover intention among healthcare workers in primary healthcare centers in Saudi Arabia and assessed the possible role of social support in reducing this association. This study is complementary to a recent study evaluating various work-related challenges affecting Healthcare workers in Saudi Arabia [17]. As a result, the main hypothesis for this study is: Social support can mediate the association between stress and turnover among healthcare workers in Saudi Arabia during the COVID-19 pandemic.

## Materials and methods

Because of the social distancing measures in Saudi Arabia, this cross-sectional study was conducted using an online questionnaire. The study population included healthcare workers aged 18 years or more and working at different primary healthcare centers in Saudi Arabia. The term healthcare workers included physicians, nurses, paramedics, and administrative workers.

### Sampling approach

To access a representative sample of healthcare workers, Saudi Arabia was divided geographically into five regions: Northern, Western, Southern, Eastern, and Central regions. The Saudi Ministry of Health is represented in the five regions. It is composed of health directorates, and each health directorate supervises a number of primary healthcare centers within its geographical region. In this study, six primary healthcare centers were randomly selected from every health directorate. Then, e-mails including links to the online questionnaire were sent to all healthcare workers in the selected primary healthcare centers on September 27, 2020, before reminders were sent a week later. A time span of three weeks was given to healthcare workers to complete their questionnaires, and the response rate was 73.4%.

The least sample size was estimated using the Epi- Info version 7 StatCalc designed by the Centers for Disease Control and Prevention (CDC) and the World Health Organization (WHO). In order to enhance statistical power and avoid an unpredicted low response rate, the least required sample size was more than doubled. Eventually, 1500 Healthcare workers were invited to participate in this study. Also, G*Power software, version 3.1.9.7, was used to calculate the power of this study, and this sample size (1101) has around 99% power (1-Beta error) at an alpha of 0.05, with two predictors and one criterion, to detect even a small effect size of 0.02 [18]. Linear multiple regression was used because it is the most similar test to the mediation within G*Power software.

### The online questionnaire

The online questionnaire was composed of four sections. The first section showed in detail the steps and aims of the study and ended with a question asking the respondents whether they agreed to participate. The second section included questions about participants’ age (years), sex (male or female), marital status (married or unmarried), citizenship (Saudi or non-Saudi), educational level (university degree and higher or high school level), smoking history (current smoker or non-current smoker), working hours per day and working days, and job title (physician, nurse, paramedic, or administrative worker). The third section included two scales; the Arabic versions of the Perceived Stress Scale-10 (PSS-10) and the Multidimensional Scale of Perceived Social Support (MSPSS).

PSS-10 is a reliable and valid scale to assess perceived global stress. This scale is composed of ten statements. Respondents have to express how much they agree with these statements on a Likert scale from zero (never) to four (very often), with higher scores indicating higher stress levels [19, 20]. The total score of stress can be interpreted as follows: low stress (0–13), moderate stress (14–26), and high stress (27–40). This study showed that this scale had good reliability (Cronbach’s alpha 0.85).

MSPSS a valid tool was to assess perceived social support from the following support resources, family, friends, and others. The scale is composed of seven statements, and respondents select a choice on a Likert scale ranging between one (very strongly disagree) and seven (very strongly agree), with higher scores indicating higher social support levels [21]. This study showed that this scale had good reliability (Cronbach’s alpha 0.92). The fourth section included one question asking healthcare workers if they intend to leave their primary healthcare centers within the next few months, and the available responses were yes and no.

### Ethical considerations

The Central Institutional Review Board of the Ministry of Health in Saudi Arabia approved the study protocol. All participating Healthcare workers were asked to select "accept to participate" on the first page and "submit answers" on the last page of the online questionnaire. The study was performed per the principles of the Declaration of Helsinki.

### Statistical analysis

The Statistical Package for Social Science (SPSS) version 23 was used for analysis, including AMOS. Descriptive analysis, including mean, standard deviation, frequencies, and percentage, was used to describe the study sample. Also, a chi-square test between the turnover and the demographic variables was used in order to advance understanding of the sample. The included variables in the chi-square test were age, sex, marital status, smoking, education, nationality, and job. Finally, path analysis using AMOS was conducted in order to evaluate how social support mediate the relationship between stress and turnover among the study sample.

## Results

According to Table 1, 1101 healthcare workers (242 physicians, 340 nurses, 310 paramedics, and 209 administrative workers) participated in this study. The participants were men (56%) and women (46%). The vast majority of the study participants were married (83.8%), and only (16.2%) were not married. The nationality of the participants revealed that (85.4%) were Saudi citizens and non-citizens were (14.6%). Most of the participants had a high school diploma (56.4%), while only (43.6%) had a university degree. Of the participants, only (19.3%) were current smokers (Table 1).

**Table 1:** Characteristics of healthcare workers.

Characteristics		n = 1101 (%)
Age (Mean±Sd) years		38.9±8.1
Sex	Men		617 (56.0)
	Women		484 (44.0)
Marital status	Married		923 (83.8)
	Others		178 (16.2)
Nationality	Saudi		940 (85.4)
	Non-Saudi		161 (14.6)
Education	University		480 (43.6)
	High school		621 (56.4)
Smoking	Current		212 (19.3)
	Non-current		889 (80.7)
Job	Physician		242 (22.0)
	Nurse		340 (30.9)
	Paramedical		310 (28.1)
	Administrative		209 (19.0)
Working hours/days (Mean±Sd)		8.19±1.2
Stress		27.3±7.5
Social support		49.86±12.68
Turnover	Yes	354 (32.2)
	No	747 (67.8)

The result of chi-square test of independence showed that there was no significant association between turnover and the following variables: age, nationality, marital status, education level, and smoke.

A chi-square test of independence showed that there was a significant association between gender and turnover, X2(1, N = 1,101) = 10.89, p < .01. Of the male participants, 173 (28.0%) were wanting to leave the work, and 444 (72.0%) were not wanting to leave the work. Of the female participants, 181 (37.4%) were wanting to leave the work, and 303 (62.6%) were not wanting to leave the work (Table 2).

**Table 2:** Demographic characteristics by turnover intention.

Variables	Turnover intention		p-value
	Yes n = 354 (%)	No n = 747 (%)	
**Age**			
18 to 30 years	44 (28.0)	113 (72.0)	p > 0.05
31 to 40 years	200 (35.3)	366 (64.7)	
41 to 50 years	79 (30.7)	178 (69.3)	
51 to 65 years	30 (25.4)	88 (74.6)	
**Gender**			p < 0.001
Male	173 (28.0)	444 (72.0)	
Female	181 (37.4)	303 (62.6)	
**Nationality**			p > 0.05
Saudi	299 (31.8)	641 (68.2)	
Non-Saudi	55 (34.2)	106 (65.8)	
**Marital status**			p > 0.05
Married	298 (32.3)	625 (67.7)	
Not Married	56 (31.5)	122 (68.5)	
**Education Level**			p > 0.05
Less than Bachelor	188 (30.3)	433 (69.7)	
Bachelor or Higher	166 (34.6)	314 (65.4)	
**Smoke**			
Yes	71 (33.5)	141 (66.5)	p > 0.05
No	283 (31.8)	606 (68.2)	
**Job**			
Physician	84 (34.7)	158 (65.3)	p < 0.001
Nurse	134 (39.4)	206 (60.6)	
Paramedical	84 (27.1)	226 (72.9)	
Administrative	52 (24.9)	157 (75.1)	

A chi-square test of independence showed that there was a significant association between job type and turnover, X2(3, N = 1,101) = 17.64, p < .01. Of the physicians, 84 (34.7%) were wanting to leave the work, and 158 (65.3%) were not wanting to leave the work. Of the nurses, 134 (39.4%) were wanting to leave the work, and 206 (60.6%) were not wanting to leave the work. Of the paramedical staff, 84 (27.1%) were wanting to leave the work, and 226 (72.9%) were not wanting to leave the work. Of the administrative staff, 52 (24.9%) were wanting to leave the work, and 157 (75.1%) were not wanting to leave the work (Table 2).

**Fig 1:**
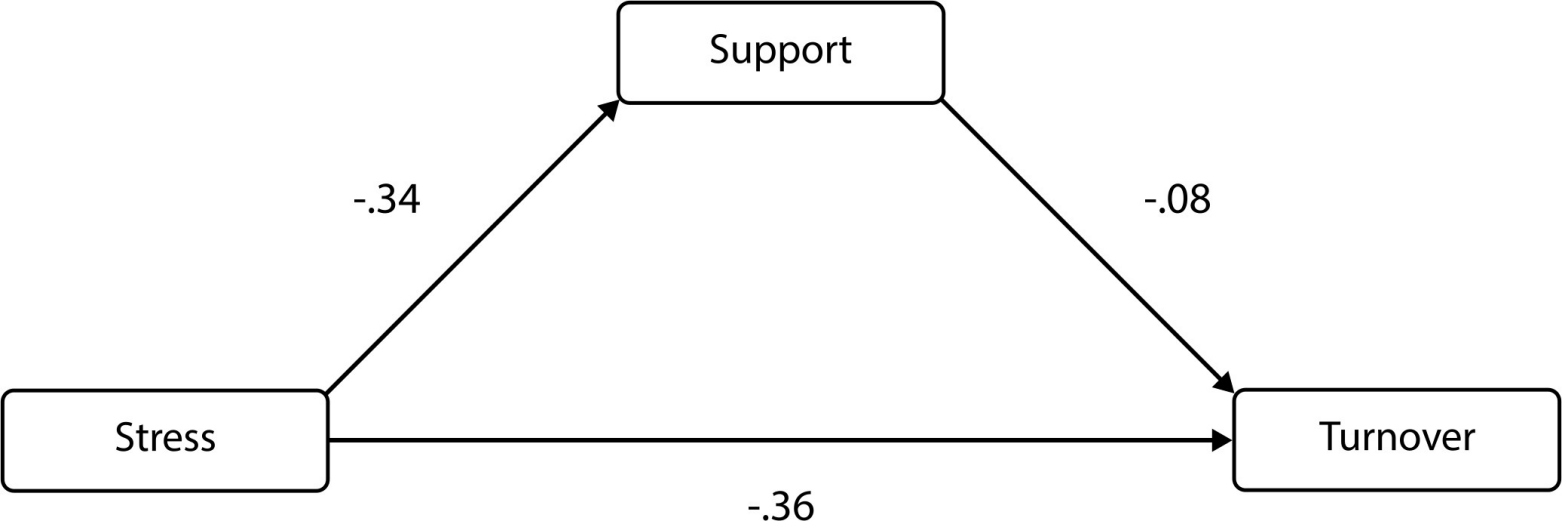
The result of path model analysis for the mediation model.

A path analysis was run to look at the mediating effect of support on the relationship between stress and turnover. The path between stress and support had a significant standardized regression weight (= -.34, p < .05). The path between support and turnover had a significant standardized regression weight (= .08, p < .05). The standardized total effect of stress on turnover without the impact of support was significant (-.39, p < .05). The direct effect of stress on the turnover with support was significant (-.36, p < .05). The indirect effect of stress on the turnover with support was significant (-.03, p < .05). Thus, there is evidence to show that support mediates the relationship between stress and support (Fig 1).

## Discussion

Stress level was found high among the healthcare workers in the primary health care centers. This result is supported by a systematic review study that found stress is a common problem among healthcare workers [9]. Also, a cross sectional study on Palestinian healthcare workers revealed that the majority of the study participants were suffer from high level of stress [8]. This study showed that levels of stress among healthcare workers in primary healthcare centers in Saudi Arabia were positively associated with turnover intention during the COVID-19 pandemic. A study in Egypt supported this result since they found the majority of the nurses had turnover intention resulting from stress [12]. Social support was shown to mitigate this association between stress and turnover. This finding is alarming because the turnover of healthcare workers may undermine the efforts of the Saudi government to control the COVID-19 pandemic. A systematic review study found stress has a negative association with social support which support the findings of current study [9]. Also, a study in China found social support is positively associated with better mental health [15].

This study had several strengths. First, it is the first study, to the best of my knowledge, to assess the relationship between stress and turnover among healthcare workers in Saudi Arabia during the COVID-19 pandemic. Secondly, the study used a multi-stage random sampling approach to recruit a large cohort of healthcare workers in primary healthcare centers in Saudi Arabia. Thirdly, this research employed validated scales to assess stress and other covariates. Fourth, the results were controlled for several sociodemographic and potential occupational confounders.

This study had several limitations. First, the cross-sectional design of this study did not imply causality. Second, due to the social distancing regulations related to the COVID-19 pandemic, healthcare workers were recruited by e-mails, and online surveys collected data. Online surveys, in general, are vulnerable to non-response bias since non-respondents might carry different sociodemographic and occupational characteristics compared to respondents [22, 23]. Third, this study did not assess COVID-19 perceptions among healthcare workers. A recent study showed that COVID-19 perceptions were tightly associated with job insecurity and burnout; thus, they may represent an important confounder [24]. Fourth, the turnover intention was assessed using a yes/no question. Using a validated scale to assess the turnover intention might have been more informative.

In conclusion, this study indicated that high stress among healthcare workers in Saudi Arabia might be associated with increased turnover intention during the COVID-19 pandemic. However, social support can mediate the association and weaken the impact of stress on turnover intention. Different types of support can be used to reduce psychological stress and consequently decrease turnover intention. As a result, the health care workers should be provided with the available resources in the community which can satisfy their needs. Social support can be used as a coping strategy to reduce stress, as supported by psychological stress theory [25] which can reduce the turnover among healthcare workers especially the Saudi population has good social ties.

## Supporting information

S1 File (PDF)

S1 Data (SAV)
