# Supplementary material for: Stress and turnover intention among healthcare workers in Saudi Arabia during the time of COVID-19: Can social support play a role?
Source: PLoS One. 2021 Oct 7;16(10):e0258101. doi: 10.1371/journal.pone.0258101 (PMC8496805; doi:10.1371/journal.pone.0258101)
Supplement: S1 File — (PDF) [file pone.0258101.s001.pdf]

C:\Users\11saa\OneDrive\سطح المكتب\Khaled Turn\Turnover22.amw

Analysis Summary

Date and Time

Date: Friday, May 21, 2021
Time: 2:29:15 PM

Title

Turnover22: Friday, May 21, 2021 2:29 PM

Groups

Group number 1 (Group number 1)

Notes for Group (Group number 1)

The model is recursive.
Sample size = 1101

Variable Summary (Group number 1)

Your model contains the following variables (Group number 1)

Observed, endogenous variables

Support
Turnover

Observed, exogenous variables

RR_Stresss

Unobserved, exogenous variables

e1
e2

Variable counts (Group number 1)

Number of variables in your model:	5
Number of observed variables:	3
Number of unobserved variables:	2
Number of exogenous variables:	3
Number of endogenous variables:	2

Labeled	0	0	0	0	0	0
Unlabeled	3	0	3	1	2	9
Total	5	0	3	1	2	11

Models

Default model (Default model)

Notes for Model (Default model)

Computation of degrees of freedom (Default model)

Number of distinct sample moments: 9
Number of distinct parameters to be estimated: 9
Degrees of freedom (9 - 9): 0

Result (Default model)

Minimum was achieved
Chi-square = .000
Degrees of freedom = 0
Probability level cannot be computed

Group number 1 (Group number 1 - Default model)

Estimates (Group number 1 - Default model)

Scalar Estimates (Group number 1 - Default model)

Maximum Likelihood Estimates

Regression Weights: (Group number 1 - Default model)

			Estimate	S.E.	C.R.	P	Label
Support	<---	RR_Stresss	-.572	.048	-12.007	***	
Turnover	<---	Support	.003	.001	2.870	.004	
Turnover	<---	RR_Stresss	-.022	.002	-12.319	***	

Standardized Regression Weights: (Group number 1 - Default model)

			Estimate
Support	<---	RR_Stresss	-.340
Turnover	<---	Support	.084
Turnover	<---	RR_Stresss	-.362

Means: (Group number 1 - Default model)

Intercepts: (Group number 1 - Default model)

	Estimate	S.E.	C.R.	P	Label
Support	65.507	1.352	48.464	***	
Turnover	2.137	.086	24.846	***	

Variances: (Group number 1 - Default model)

	Estimate	S.E.	C.R.	P	Label
RR_Stresss	56.808	2.422	23.452	***	
e1	142.047	6.057	23.452	***	
e2	.183	.008	23.452	***	

Matrices (Group number 1 - Default model)

Total Effects (Group number 1 - Default model)

	RR_Stresss	Support
Support	-.572	.000
Turnover	-.024	.003

Standardized Total Effects (Group number 1 - Default model)

	RR_Stresss	Support
Support	-.340	.000
Turnover	-.391	.084

Direct Effects (Group number 1 - Default model)

	RR_Stresss	Support
Support	-.572	.000
Turnover	-.022	.003

Standardized Direct Effects (Group number 1 - Default model)

	RR_Stresss	Support
Support	-.340	.000
Turnover	-.362	.084

Indirect Effects (Group number 1 - Default model)

	RR_Stresss	Support
Support	.000	.000
Turnover	-.002	.000

Support	.000	.000
Turnover	-.029	.000

Bootstrap (Group number 1 - Default model)

Bootstrap standard errors (Group number 1 - Default model)

Scalar Estimates (Group number 1 - Default model)

Regression Weights: (Group number 1 - Default model)

Parameter			SE	SE-SE	Mean	Bias	SE-Bias
Support	<---	RR_Stresss	.053	.002	-.574	-.002	.002
Turnover	<---	Support	.001	.000	.003	.000	.000
Turnover	<---	RR_Stresss	.002	.000	-.022	.000	.000

Standardized Regression Weights: (Group number 1 - Default model)

Parameter			SE	SE-SE	Mean	Bias	SE-Bias
Support	<---	RR_Stresss	.028	.001	-.341	-.001	.001
Turnover	<---	Support	.030	.001	.085	.000	.001
Turnover	<---	RR_Stresss	.027	.001	-.361	.001	.001

Means: (Group number 1 - Default model)

Parameter	SE	SE-SE	Mean	Bias	SE-Bias
RR_Stresss	.226	.007	27.325	-.005	.010

Intercepts: (Group number 1 - Default model)

Parameter	SE	SE-SE	Mean	Bias	SE-Bias
Support	1.377	.044	65.567	.061	.062
Turnover	.087	.003	2.135	-.002	.004

Variances: (Group number 1 - Default model)

Parameter	SE	SE-SE	Mean	Bias	SE-Bias
RR_Stresss	2.243	.071	56.679	-.129	.100
e1	6.544	.207	141.619	-.428	.293
e2	.006	.000	.183	.000	.000

Matrices (Group number 1 - Default model)

Total Effects - Standard Errors (Group number 1 - Default model)

Standardized Total Effects - Standard Errors (Group number 1 - Default model)

	RR_Stresss	Support
Support	.028	.000
Turnover	.024	.030

Direct Effects - Standard Errors (Group number 1 - Default model)

	RR_Stresss	Support
Support	.053	.000
Turnover	.002	.001

Standardized Direct Effects - Standard Errors (Group number 1 - Default model)

	RR_Stresss	Support
Support	.028	.000
Turnover	.027	.030

Indirect Effects - Standard Errors (Group number 1 - Default model)

	RR_Stresss	Support
Support	.000	.000
Turnover	.001	.000

Standardized Indirect Effects - Standard Errors (Group number 1 - Default model)

	RR_Stresss	Support
Support	.000	.000
Turnover	.011	.000

Bootstrap Confidence (Group number 1 - Default model)

Bias-corrected percentile method (Group number 1 - Default model)

95% confidence intervals (bias-corrected percentile method)

Scalar Estimates (Group number 1 - Default model)

Regression Weights: (Group number 1 - Default model)

Parameter			Estimate	Lower	Upper	P
Support	<---	RR_Stresss	-.572	-.679	-.468	.005
Turnover	<---	Support	.003	.001	.005	.014
Turnover	<---	RR_Stresss	-.022	-.026	-.019	.003

Turnover <---	Support	.084	.019	.139	.015
Turnover <---	RR_Stresss	-.362	-.421	-.314	.002

Means: (Group number 1 - Default model)

Parameter	Estimate	Lower	Upper	P
RR_Stresss	27.330	26.939	27.782	.003

Intercepts: (Group number 1 - Default model)

Parameter	Estimate	Lower	Upper	P
Support	65.507	62.891	68.204	.005
Turnover	2.137	1.969	2.313	.003

Variances: (Group number 1 - Default model)

Parameter	Estimate	Lower	Upper	P
RR_Stresss	56.808	52.637	61.609	.002
e1	142.047	131.459	157.286	.002
e2	.183	.172	.194	.003

Matrices (Group number 1 - Default model)

Total Effects (Group number 1 - Default model)

Total Effects - Lower Bounds (BC) (Group number 1 - Default model)

	RR_Stresss	Support
Support	-.679	.000
Turnover	-.027	.001

Total Effects - Upper Bounds (BC) (Group number 1 - Default model)

	RR_Stresss	Support
Support	-.468	.000
Turnover	-.021	.005

Total Effects - Two Tailed Significance (BC) (Group number 1 - Default model)

	RR_Stresss	Support
Support	.005	...
Turnover	.004	.014

Standardized Total Effects (Group number 1 - Default model)

	RR_Stresss	Support
Support	-.402	.000
Turnover	-.437	.019

Standardized Total Effects - Upper Bounds (BC) (Group number 1 - Default model)

	RR_Stresss	Support
Support	-.287	.000
Turnover	-.345	.139

Standardized Total Effects - Two Tailed Significance (BC) (Group number 1 - Default model)

	RR_Stresss	Support
Support	.004	...
Turnover	.004	.015

Direct Effects (Group number 1 - Default model)

Direct Effects - Lower Bounds (BC) (Group number 1 - Default model)

	RR_Stresss	Support
Support	-.679	.000
Turnover	-.026	.001

Direct Effects - Upper Bounds (BC) (Group number 1 - Default model)

	RR_Stresss	Support
Support	-.468	.000
Turnover	-.019	.005

Direct Effects - Two Tailed Significance (BC) (Group number 1 - Default model)

	RR_Stresss	Support
Support	.005	...
Turnover	.003	.014

Standardized Direct Effects (Group number 1 - Default model)

Standardized Direct Effects - Lower Bounds (BC) (Group number 1 - Default model)

	RR_Stresss	Support
Support	-.402	.000
Turnover	-.421	.019

Standardized Direct Effects - Upper Bounds (BC) (Group number 1 - Default model)

Standardized Direct Effects - Two Tailed Significance (BC) (Group number 1 - Default model)

	RR_Stresss	Support
Support	.004	...
Turnover	.002	.015

Indirect Effects (Group number 1 - Default model)

Indirect Effects - Lower Bounds (BC) (Group number 1 - Default model)

	RR_Stresss	Support
Support	.000	.000
Turnover	-.003	.000

Indirect Effects - Upper Bounds (BC) (Group number 1 - Default model)

	RR_Stresss	Support
Support	.000	.000
Turnover	.000	.000

Indirect Effects - Two Tailed Significance (BC) (Group number 1 - Default model)

	RR_Stresss	Support
Support	...	...
Turnover	.015	...

Standardized Indirect Effects (Group number 1 - Default model)

Standardized Indirect Effects - Lower Bounds (BC) (Group number 1 - Default model)

	RR_Stresss	Support
Support	.000	.000
Turnover	-.051	.000

Standardized Indirect Effects - Upper Bounds (BC) (Group number 1 - Default model)

	RR_Stresss	Support
Support	.000	.000
Turnover	-.006	.000

Standardized Indirect Effects - Two Tailed Significance (BC) (Group number 1 - Default model)

	RR_Stresss	Support
Support	...	...
Turnover	.014	[illegible]

Iteration		Negative eigenvalues	Condition #	Smallest eigenvalue	Diameter	F	NTries	
0	e	0	250.419		9999.000	1240.135	0	999
1	e	0	270.114		1.071	930.046	2	
2	e	0	214.214		.195	340.081	1	
3	e	0	206.927		.153	101.503	1	
4	e	0	203.897		.140	18.619	1	
5	e	0	207.132		.099	1.234	1	
6	e	0	207.898		.035	.009	1	
7	e	0	206.975		.003	.000	1	
8	e	0	205.632		.000	.000	1	

Bootstrap (Default model)

Summary of Bootstrap Iterations (Default model)

(Default model)

Iterations	Method 0	Method 1	Method 2
1	0	0	0
2	0	0	0
3	0	2	0
4	0	171	0
5	0	307	0
6	0	20	0
7	0	0	0
8	0	0	0
9	0	0	0
10	0	0	0
11	0	0	0
12	0	0	0
13	0	0	0
14	0	0	0
15	0	0	0
16	0	0	0
17	0	0	0
18	0	0	0
19	0	0	0
Total	0	500	0

0 bootstrap samples were unused because of a singular covariance matrix.
0 bootstrap samples were unused because a solution was not found.
500 usable bootstrap samples were obtained.

[illegible]

```
                          .000    |********************
                          .000    |
                          .000    |
                          .000    |
                          .000    |
                          .000    |
                          .000    |
  N = 500                 .000    |
  Mean = .000             .000    |
  S. e. = .000            .000    |
                          .000    |
                          .000    |
                          .000    |
                          .000    |
                          .000    |
                                  |--------------------
```

ML discrepancy (implied vs pop) (Default model)

```
                                  |--------------------
                          .860    |**
                         3.415    |************
                         5.970    |*******************
                         8.525    |*******************
                        11.080    |**********
                        13.635    |********
                        16.190    |***
  N = 500               18.746    |**
  Mean = 8.502          21.301    |*
  S. e. = .191          23.856    |*
                        26.411    |
                        28.966    |
                        31.521    |
                        34.076    |
                        36.632    |*
                                  |--------------------
```

K-L overoptimism (unstabilized) (Default model)

```
                                  |--------------------
                      -191.626    |*
                      -161.463    |*
                      -131.301    |***
```

```
                      -10.651   |*****************
N = 500                19.512   |**************
Mean = 17.925          49.674   |***************
S. e. = 3.436          79.837   |*************
                      109.999   |********
                      140.162   |*****
                      170.324   |**
                      200.487   |*
                      230.649   |*
                                |--------------------
```

K-L overoptimism (stabilized) (Default model)

```
                              |--------------------
                      6.801   |****
                     10.013   |**************
                     13.226   |********************
                     16.438   |*****************
                     19.651   |************
                     22.863   |*********
                     26.076   |****
N = 500              29.288   |***
Mean = 17.116        32.500   |***
S. e. = .302         35.713   |*
                     38.925   |*
                     42.138   |
                     45.350   |*
                     48.562   |*
                     51.775   |*
                              |--------------------
```

Model Fit Summary

CMIN

Model	NPAR	CMIN	DF	P	CMIN/DF
Default model	9	.000	0		
Saturated model	9	.000	0		
Independence model	6	326.144	3	.000	108.715

Baseline Comparisons

Model	NFI [illegible]	RFI [illegible]	IFI [illegible]	TLI [illegible]	CFI

Parsimony-Adjusted Measures

Model	PRATIO	PNFI	PCFI
Default model	.000	.000	.000
Saturated model	.000	.000	.000
Independence model	1.000	.000	.000

NCP

Model	NCP	LO 90	HI 90
Default model	.000	.000	.000
Saturated model	.000	.000	.000
Independence model	323.144	267.526	386.170

FMIN

Model	FMIN	F0	LO 90	HI 90
Default model	.000	.000	.000	.000
Saturated model	.000	.000	.000	.000
Independence model	.296	.294	.243	.351

RMSEA

Model	RMSEA	LO 90	HI 90	PCLOSE
Independence model	.313	.285	.342	.000

AIC

Model	AIC	BCC	BIC	CAIC
Default model	18.000	18.066		
Saturated model	18.000	18.066		
Independence model	338.144	338.188		

ECVI

Model	ECVI	LO 90	HI 90	MECVI
Default model	.016	.016	.016	.016
Saturated model	.016	.016	.016	.016
Independence model	.307	.257	.365	.307

HOELTER

Model	HOELTER .05	HOELTER .01

Minimization:	.049
Miscellaneous:	.342
Bootstrap:	.225
Total:	.616
